# Efavirenz: History, Development and Future

**DOI:** 10.3390/biom13010088

**Published:** 2022-12-31

**Authors:** Bárbara Costa, Nuno Vale

**Affiliations:** 1 OncoPharma Research Group, Center for Health Technology and Services Research (CINTESIS), Rua Doutor Plácido da Costa, 4200-450 Porto, Portugal; 2 CINTESIS@RISE, Faculty of Medicine, University of Porto, Alameda Professor Hernâni Monteiro, 4200-319 Porto, Portugal; 3 Department of Community Medicine, Information and Health Decision Sciences (MEDCIDS), Faculty of Medicine, University of Porto, Rua Doutor Plácido da Costa, 4200-450 Porto, Portugal

**Keywords:** efavirenz, NNRTI, HIV, antiviral therapy, clinical trials

## Abstract

Efavirenz (Sustiva^®^) is a first-generation non-nucleoside reverse transcriptase inhibitor (NNRTI) used to treat human immunodeficiency virus (HIV) type 1 infection or to prevent the spread of HIV. In 1998, the FDA authorized efavirenz for the treatment of HIV-1 infection. Patients formerly required three 200 mg efavirenz capsules daily, which was rapidly updated to a 600 mg tablet that only required one tablet per day. However, when given 600 mg once daily, plasma efavirenz concentrations were linked not only to poor HIV suppression but also to toxicity. Clinical data suggested that the standard dose of efavirenz could be reduced without compromising its effectiveness, resulting in a reduction in side effects and making the drug more affordable. Therefore, ENCORE1 was performed to compare the efficiency and safeness of a reduced dose of efavirenz (400 mg) with the standard dose (600 mg) plus two NRTI in antiretroviral-naïve HIV-infected individuals. Nowadays, due to the emergence of integrase strand transfer inhibitors (INSTIs), some consider that it is time to stop using efavirenz as a first-line treatment on a global scale, in the parts of the world where that is possible. Efavirenz has been a primary first-line antiviral drug for more than 15 years. However, at this moment, the best use for efavirenz could be for pre-exposure prophylaxis (PrEP) and repurposing in medicine.

## 1. History

**Figure 1:**
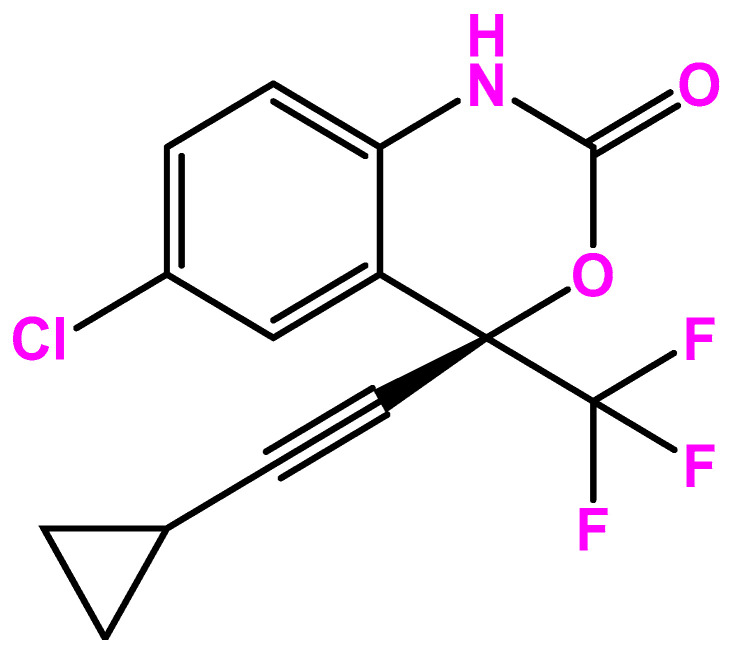
Efavirenz chemical structure.

Efavirenz is the first-generation non-nucleoside reverse transcriptase inhibitor (NNRTI) used to treat immunodeficiency virus (HIV) type 1 infection or prevent the spread of HIV. It is sold under the brand names Sustiva^®^ and Stocrin^®^ and is the lead compound of a series of benzoxazinones developed initially by DuPont Pharmaceuticals (Figure 1). In 1997, clinical studies started to assess the efficacy of the triple combination of efavirenz with nelfinavir, indinavir, ritonavir, or other retroviral for the treatment of opportunistic and pediatric viral infections. Then, the effectiveness of efavirenz was studied both alone and in combination with zidovudine and lamivudine. Later, a study involving eight HIV-positive patients was presented at the 12th World AIDS Conference in July 1998, demonstrating that the administration of efavirenz in dual and triple combinations reduced the level of detectable HIV-RNA in plasma [1].

The Food and Drug Administration (FDA) authorized efavirenz on 7 June 1998, with dose of 600 mg orally once-daily (200 mg × 3 capsules, once-daily) for the treatment of HIV infection, and in the European Union in 1999. On 17 February 2016, FDA gave its approval for the generic tablet formulation to Mylan Pharmaceuticals. After getting WHO clearance, Thailand’s Government Pharmaceutical Organization (GPO) declared that it would begin manufacturing efavirenz in late 2018. Today, this drug is registered on the World Health Organization’s (WHO) list of essential medicines for priority diseases [2]. Efavirenz was one of the first acquired immunodeficiency syndrome (AIDS) medications to be authorized for once-daily use. Patients formerly required three 200 mg efavirenz capsules daily, which was later updated to a 600 mg tablet that only required one tablet per day [3,4]. However, in 2001, when given 600 mg once daily, plasma efavirenz concentrations were linked not only to poor HIV suppression but also to toxicity [5]. Numerous investigations followed this association and discovered that a high efavirenz concentration was linked to unfavorable occurrences such as neuropsychiatric symptoms and poor liver function [6,7]. Because of its tolerance profile and the discovery of integrase inhibitors, efavirenz is no longer regarded as a recommended medication in developed countries. It is now referred to as an alternative therapy option [8,9].

These findings have given rise to discussion on the ideal efavirenz dosage. In specific circumstances, such as when cytochrome P450 polymorphism is present (which has been linked to greater plasma efavirenz concentrations), some studies have recommended lowering the dose of efavirenz [10]. This, however, is not practical in actual practice due to the expense and logistical challenges of wide genotyping. It was when Carey et al. performed a study regarding the safety and efficacy of reduced versus standard dose efavirenz (EFV) plus two nucleotide reverse transcriptase inhibitors in antiretroviral-naive HIV-infected individuals (ENCORE1 study, NCT01011413) found that the standard dose of efavirenz could be scaled back without losing effectiveness. The 400 mg dose maintained similar viral suppression, reduced side effects, and made this drug more affordable [11]. ENCORE 1 established that the efficacy and tolerance of efavirenz 400 mg daily (for viral control at week 48) was noninferior to efavirenz 600 mg daily as initial therapy for HIV treatment [11,12]. Moreover, ENCORE1 allowed researchers to investigate these variables in a geographically and genetically varied patient population and to investigate connections between efficacy and safety outcomes with low dose efavirenz.

The barrier to HIV resistance is relatively low for NNRTIs currently on the market and the second generation of NNRTIs, efavirenz, nevirapine, delviradine, and rilpivirine [13]. All members of this class, with the exception of etravirine, are susceptible to single-point RT alterations that would make them inactive [14]. Due to the low resistance barrier, NNRTIs are typically used early in therapy. This is when HIV resistance to these drugs is least likely, and the combined protective effect of three fully active drugs is most potent [15,16]. The initial therapeutic regimen, which includes one or two NRTIs, protease inhibitors (PI), and NNRTIs, is crucial. Nevirapine or efavirenz are widely used in the initial regimen in developing nations due to their efficacy, low cost, and convenient dosage schedule [16].

### 1.1. Pharmacodynamics

Efavirenz inhibits the activity of viral RNA-directed DNA polymerase (i.e., reverse transcriptase) [17]. The antiviral activity of efavirenz is dependent on intracellular conversion to the active triphosphorylated form. The rate of efavirenz phosphorylation varies, depending on cell type. It is believed that inhibition of reverse transcriptase interferes with the generation of DNA copies of viral RNA, which, in turn, are necessary for the synthesis of new virions. Intracellular enzymes subsequently eliminate the HIV particle (or even the intact particles) that previously had been uncoated and left unprotected during entry into the host cell. Thus, reverse transcriptase inhibitors are virustatic and do not eliminate HIV from the body. Even though human DNA polymerase is less susceptible to the pharmacologic effects of triphosphorylated efavirenz, this action may nevertheless account for some of the drug’s toxicity [18].

#### 1.1.1. Anti-HIV Effects

Efavirenz falls in the NNRTI class of antiretrovirals. Both nucleoside and non-nucleoside RTIs inhibit the same target, the reverse transcriptase enzyme, an essential viral enzyme that transcribes viral RNA into DNA. Unlike nucleoside RTIs (NRTIs), which bind at the enzyme’s active site, NNRTIs act allosterically by binding to a distinct site away from the active site known as the NNRTI pocket [19]. Efavirenz is ineffective against HIV-2, as the pocket of the HIV-2 reverse transcriptase has a different structure, which confers intrinsic resistance to the NNRTI class [20].

As most NNRTIs bind within the same pocket, viral strains which are resistant to efavirenz are usually also resistant to the other NNRTIs. The most common mutation observed after efavirenz treatment is K103N. NRTIs and efavirenz have different binding targets, so cross-resistance is unlikely; the same is true about efavirenz and protease inhibitors [21].

#### 1.1.2. Neuropsychiatric Effects

Efavirenz appears to cause neuropsychiatric side effects in approximately 50% of patients. These effects typically start soon after the beginning of medication and frequently peak two weeks later. They can range from depression, anxiety, and sleep issues to more aggressive behavior, paranoia, and psychosis [22]. Neuropsychiatric effects frequently have a detrimental effect on treatment adherence. It is well known that some factors can raise the possibility of neuropsychiatric side effects in HIV-positive patients, such as weight, gender and CYP450 2B6 [23]. The behavioral effects of efavirenz seem to be dose-dependent, mainly mediated by the 5-HT2A receptor, which is the lysergic acid diethylamide main site of action (LSD) [24]. Furthermore, it may be challenging to discern between the neuropsychiatric side effects of efavirenz and those caused by substance abuse, preexisting mental illness, or HIV-related neuropsychiatric symptoms. Although the adverse events are dose-dependent, they are often reversible [25].

### 1.2. Pharmacokinetics

Peak plasma concentrations take between 3 and 5 h to reach. Following a 600 mg average adult oral dose, EFV is easily absorbed and reaches a peak serum concentration (Cmax) of 4.07 mcg/quantities or doses of 200, 400, and 600 mg EFV, increases in Cmax and the area under the plasma concentration-time curve (AUC) are dosage proportional [26]. EFV has a lengthy serum half-life of 45 h and takes 6 to 10 days to attain steady-state plasma concentrations. When compared to fasting, a reduced-fat/normal-calorie meal (EFV capsules) and, in particular, a high-fat/high-calorie meal (EFV capsules and tablets), both increase the bioavailability of EFV.

The cytochrome P450 system principally metabolizes efavirenz to hydroxylated metabolites with subsequent glucuronidation of these hydroxylated metabolites. Metabolized efavirenz forms 7-hydroxy and 8-hydroxy efavirenz (8-OH-efavirenz is the main metabolite), and the formation rate of this metabolism has been proven to have different variability between human microsome samples [27]. However, these metabolites are essentially inactive against HIV-1. The main metabolite of EFV detected in urine is 8-hydroxy-EFV-gluc various UDP-glucuronosyltransferase (UGT) isoforms can process the hydroxylated EFV metabolites processed by various UDP-glucuronosyltransferase (UGT) isoforms to create glucuronide form [28]. The rate at which EFV-N—glucuronide forms varies greatly among human microsome samples as well.

The efavirenz pharmacokinetics are characterized by significant between-subject variability, which influences both therapeutic response and adverse effects. Genetic variation in cytochrome P450 genes, especially in CYP2A6, has been linked to some of the variability in efavirenz pharmacokinetics. After stopping an EFV-based regimen, the CYP2B6 G516T polymorphism has also been linked to a prolonged elimination serum half-life and an increased risk of developing drug resistance [29]. The likelihood ratio of having very high EFV plasma levels was 35 (95% CI, 11–110) in people with a poor metabolizer genotype. The genotypes of CYP2B6 poor metabolizers can identify those who are at risk of having high plasma concentrations of EFV. High EFV plasma concentrations and effective EFV dosage reduction based on genotype. The high prevalence of EFV-related CYP2B6 polymorphisms in individuals of African origin, who today make up the bulk of HIV-infected people globally, is highly significant.

#### Drug Interactions and Resistance

For currently available NNRTIs and the second generation of NNRTIs (includes: efavirenz, nevirapine, delviradine, and rilpivirine), the barrier to HIV resistance is rather low. Except for etravirine, all members of this class can be rendered inactive by single-point changes in RT. NNRTIs are frequently used early in therapy because of this low resistance barrier. This is when HIV resistance to these medications is least likely and the combined protective effect of three fully active drugs is strongest. Along with one or two NRTIs, a PI, and NNRTIs, the initial therapy regimen is essential. Due to their effectiveness, low cost, and practical dosing schedule, nevirapine or efavirenz are frequently used in the first regimen in underdeveloped nations.

Although rarely used in combination with other antiretrovirals nowadays, efavirenz alters the levels of most protease inhibitors and usually requires adjusted dosing. Efavirenz is not administered with the other NNRTI drugs due to elevated side effect risk. Drug interactions are problematic and more common among NNRTIs than NRTIs due to the significant CYP450 enzyme system’s involvement in the metabolism of medicines in this class [30]. Although to a lesser amount, efavirenz inhibits CYP2C9 and CYP2C19 and increases the hepatic CYP3A4 enzyme. In addition to CYP3A4. NNRTIs may alter the metabolism of coadministered medications that are processed by the CYP450 system, lowering (efavirenz) or raising (for example, delviradine), the plasma levels of those medications. Like this, medications that stimulate or inhibit CYP450 activity might affect how much NNRTI is present in the blood. Efavirenz also has direct interactions with ATP-binding cassette (ABC) family members, which reduces the functionality of ABC [31]

As with all other anti-HIV drugs, strains of HIV that are resistant to efavirenz may be transmitted or may emerge after a period of treatment. The emergence of drug-resistant strains coincides with a fall in the effectiveness of the drug. Even small amounts of the transmitted efavirenz-resistant virus may also restrict the drug’s effectiveness. If blood levels of the drug fall too low, this will help the development of resistance to efavirenz and may affect future treatment options [32].

Efavirenz was approved initially specifically for the treatment of HIV infections in patients who failed therapy with zidovudine. The CDC recommends that Efavirenz be given as part of a three-drug regimen that includes another nucleoside reverse transcriptase inhibitor (e.g., lamivudine, stavudine, zidovudine) and a protease inhibitor or efavirenz when treating HIV infection. Notice that efavirenz is not active against HIV-2, and recent studies have shown that the integrase inhibitor dolutegravir is more effective than efavirenz and that the NNRTI rilpivirine is better tolerated (although less effective than efavirenz in people with high viral load, above 100,000 copies/mL) [33,34].

The amount of EFV an individual has to take to reach therapeutic levels may be determined by their genotype (and a variety of other factors). In patients with the CYP2B6 slow metabolizer genotype, individualizing EFV dosages for HIV treatment may help minimize EFV exposure and CNS damage [23]. However, genotype-based dose adjustment should be because considering the fact that EFV is frequently combined with other anti-retroviral medications in a fixed dose regimen for HAART [18].

## 2. Development

### 2.1. A Comprehensive Evaluation and a Change in the Paradigm of Efavirenz 400 and 600 mg Once Daily

Clinical data suggested that the standard dose of efavirenz could be reduced without compromising its effectiveness, resulting in a reduction in side effects, and making the drug more affordable. Therefore, a randomized, double-blind, placebo-controlled clinical trial was performed to compare the efficiency and safeness of a reduced dose of efavirenz (400 mg) with the standard dose (600 mg) plus two NRTI in antiretroviral naïve HIV infected individuals who have not received any treatment, over 96 weeks [35].

ENCORE1 assessed the effectiveness and safety of tenofovir/emtricitabine (TDF/FTC) and reduced versus regular dose efavirenz (EFV) as first-line HIV therapy. At 48 weeks, the initial study revealed that 400 mg of EFV was safe and virologically superior to 600 mg [36]. The persistence of efficacy and safety is examined over 96 weeks in this investigation. At week 96, non-inferiority between EFV 400 mg and EFV 600 mg when used in conjunction with TDF/FTC as the first HIV therapy was established. Both doses showed comparable safety characteristics. These findings support the regular use of a lower EFV dose in HIV care. It also assessed patient demographics and genetic polymorphisms (CYP2B6, CYP2A6, CYP3A4, NR1I3) [37] as covariates, evaluating EFV400 and EFV600 pharmacokinetics (NONMEM v. 7.2) and examining relationships with efficacy (plasma HIV-RNA (pVL) 200 copies/mL) and safety outcomes at 48 weeks in 606 randomized ENCORE1 patients [35,38]. EFV400 was linked to a better safety profile among adults who had not received ART and to a lower rate of discontinuation while remaining effective in pregnant patients and patients with tuberculosis without adjusting the dose.

**Figure 2:**
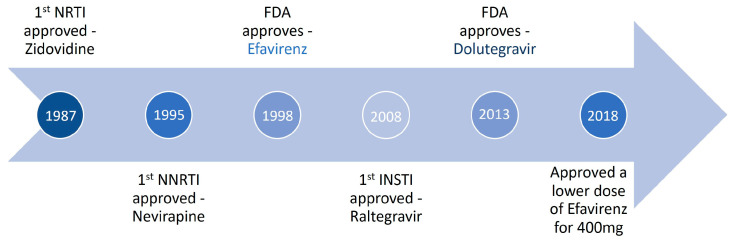
Timeline of efavirenz use to treat HIV infection.

Up to June 2018, the World Health Organization’s preferred first-line therapy for HIV-1 infection was an efavirenz-based regimen (with a 400 mg dose of efavirenz), see Figure 2. The dolutegravir-based regimen is the preferred first-line treatment, and low-dose efavirenz-based is an alternative for HIV-1 [39]. Dolutegravir is an integrase inhibitor with a better profile regarding sustained viral suppression and immunologic recovery than the EFV600-based regimen [40]. Comparative efficacy, tolerability, and safety between dolutegravir and EFV400-based regimen argue in favor of low-dose efavirenz-based regimens as an alternative to dolutegravir in combination with lamivudine/emtricitabine and tenofovir disoproxil fumarate as the preferred first-line treatment [33].

Due to adverse neurosensory effects, EFV600 has now been reduced in the most recent international recommendations. Additionally, the low genetic barrier of efavirenz can lead to the accumulation of drug-resistance mutations without comedication administration, particularly in the context of recurrent drug shortages and restricted access to routine HIV-1 RNA monitoring [41,42]. Increased mortality, the propagation of treatment-resistant mutations in HIV-1, and a rise in the prevalence of primary drug resistance are consequences of a high dose of efavirenz.

### 2.2. Human Immunodeficiency Virus Treatment Regimens Paradigm

The incidence of NNRTI resistance mutations in antiretroviral-naive individuals and the low genetic barrier of NNRTIs for developing drug resistance are both significant drawbacks of currently available NNRTIs [43,44]. Treatment failure of first-line NNRTI-based regimens can have severe repercussions, such as the further accumulation of NNRTI and nucleoside reverse transcriptase inhibitor resistance mutations [45]. This may lead to cross-resistance to second-generation NNRTIs (such as etravirine and rilpivirine) and decreased efficacy of the nucleoside “backbone” of subsequent treatment regimens, respectively [46]. Subpar immunological recovery and increased morbidity and mortality linked to virological control are possible additional effects, particularly in patients presenting advanced HIV infection [47].

Some academics even consider that it is time to stop using efavirenz as a first-line treatment on a global scale due to the emergence of integrase strand transfer inhibitors (INSTIs) that the standard of care for the management of HIV infection in some parts of the world. On the other parts, NNRTIs keep on being used as a first-line treatment due to cost and availability. Efavirenz has been a primary first-line antiviral drug for more than 15 years. Due to the increased degree of transferred NNRTI resistance in treatment-naive patients with HIV and the frequency of adverse events, efavirenz is no longer recommended as a first-line HIV treatment option in the majority of resource-rich nations. However, due to its superior safety profile compared to standard dose EFV (600 mg), low-dose EFV (400 mg) continues to be one of the first-line options for WHO guidelines. TDF/FTC/longevity EFVs of effectiveness and its comparison to INSTI regimens demonstrate the potential utility of this regimen.

The primary justifications for switching from EFV-based regimens to INSTI-based regimens in settings with limited resources are an emergent virologic failure or a contraindication to EFV (such as an increased risk of coronary heart disease or a history of neuropsychiatric conditions). However, if the world is to achieve the target of reducing HIV transmission and lowering the number of HIV-positive people by 2030, quick virologic control cannot be understated. In conclusion, the mounting data points to INSTI as the initial therapy option with the best long-term clinical efficacy, virologic control, and safety profile [33,48]

## 3. Future

### 3.1. Pre-Exposure Prophylaxis (PrEP) with Efavirenz

PrEP is one of the Joint United Nations Programme on HIV/AIDS’s five “pillars” for preventing the spread of HIV. Currently, Truvada (Emtricitabine/tenofovir) is the only drug approved for PrEP. Duwal et al. sought to investigate if efavirenz (EFV) could serve as a cheaper alternative to PrEP [49]. Using information from the ENCORE1 trial, the researcher created a population pharmacokinetic model. They managed to improve modeling for metabolic autoinduction and discover that plasma protein binding plays a significant role in the mechanisms of EFV cellular uptake. The preventive effect of various EFV dose plans following HIV exposure was then estimated using a stochastic modeling methodology.

The results demonstrated that once-daily EFV as PrEP provided 99% protection against wild-type virus if more than 50% of doses were taken. They were predicting that 400 mg oral EFV may provide superior protection against wild-type HIV. However, more research is needed to evaluate EFV as a more cost-efficient option than Truvada. Predicted prophylactic concentrations may guide the release kinetics of EFV long-acting formulations for clinical trial design.

It is important to note that this study has some limitations, recognized by the authors, such as the simplistic criterium of selecting drug candidates ignores the drug’s pharmacology, might choose only drugs that are not extensively protein bound, or select highly protein-bound candidates merely as a function of genital albumin concentration (it is unclear after how many dosing events this equilibrium between plasma and target site concentrations is achieved) [50]. Moreover, the PK model’s parametrization is based on HIV-infected individuals’ data, while prophylaxis is intended for healthy individuals [49].

Regarding efavirenz’s high potential for pharmacological interactions with potential co-medications, due to inducing numerous CYP enzymes, for example herbal remedies that may compete with CYP2B6 metabolisms to increase plasma levels, to lethal levels [51,52]. As a result, co-medication with EFV-based PrEP may necessitate caution. In any case, more clinical research is needed to determine the toxicity of EFV-PrEP and greater dose reductions would be appropriate for PrEP in specific populations. The current research offers a solid foundation to support these choices (based on the concentration-prophylaxis profiles). However, further analysis is necessary to emphasize the safety of efavirenz in the context of PrEP.

### 3.2. Repurposing Efavirenz

It is well known that the high attrition rates, lengthy drug discovery, and high costs of clinical trials highlight the need for alternate approach to identify effective therapeutic agents quickly gents. Nowadays, the identification of potential repurposed drug candidates can be suggested using a variety of data-driven and experimental methodologies. Researchers are encouraged to evaluate these medications on patients as soon as possible because most licensed treatments have several targets that are intimately tied to other disorders. Therefore, in the case of efavirenz, there is growing evidence that efavirenz could be used to treat several other diseases (see Table 1).

There is much research in favor of using efavirenz as a cancer treatment. Efavirenz slows the growth of various cancers in culture, including colorectal, pancreatic, lung, glioblastoma, and leukemia. Interestingly, this medication has been demonstrated to enhance the effects of radiation therapy in addition to being successful as a single treatment. Several clinical trials have already been performed to assess the efficacy of efavirenz, either alone or in combination with other therapies; see Table 1.

However, not all types of cancers could benefit from this drug. Efavirenz has the potential to bind to estrogen receptors with a high affinity, which could lead to breast cancer [53]. Nevertheless, patients with triple-negative breast cancer (TNBC) frequently have few pharmacological treatment options, in contrast to hormone receptor-driven breast cancer, and efavirenz has been found to be a promising anticancer treatment for treating prostate and pancreatic cancers. For these cancers, it works by inhibiting the abnormally overexpressed long interspersed nuclear element 1 (LINE-1) RT. A recent study assessed the effect of efavirenz on various TNBC cell lines. The results demonstrated that efavirenz causes cell death (inhibiting cell growth) and alters cell shape to an epithelial-like phenotype in a variety of TNBC cell lines. Furthermore, to acknowledge the fatty acid metabolism route as a crucial regulator in this efavirenz-induced anticancer process [54].

Besides cancer treatment, efavirenz can also be used for neurological disorders or other viral infectious diseases. For example, as a cholesterol-targeting drug for Alzheimer’s disease. Since the central nervous system enzyme cytochrome P450 46A1 (CYP46A1) can be activated in small doses by the reverse transcriptase inhibitor, which may aid the brain in metabolizing increasing levels of cholesterol during Alzheimer’s [55] Plus, for the potential treatment of the Zika virus (ZIKV), the authors used Vero cells to assay the ability of efavirenz and other compounds to inhibit ZIKV 2 h after infection.

**Table 1:** List of diseases where it has been studied the efficacy of efavirenz as a repurposed drug. Some of these drugs reached clinical trials and are identified by the ClinicalTrials.gov Identifier.

Disease	Target	Single or Combination Treatment	Model	ClinicalTrials.gov Identifier	Ref
Alzheimer	CYP46A1	Single	-	NCT03706885	[56]
Colorectal cancer	In vitro approach	Combination	In vivo	-	[57]
Pancreatic cancer	Activating phosphorylation of the tumor suppressor protein p53	As single treatment or in combination with radiation therapy	-	NCT00964171	[58,59]
Prostate cancer	Prostate-specific antigen (PSA) nonprogression rate	Single		NCT00964002	[60]
Lung cancer	In vitro approach (MRC-5 and A549 lung cells)	Single	In vitro	-	[61]
Glioblastoma	CYP46A1/24OHC axis, a potential therapeutic target	Single	In vitro	-	[62]
Leukemia	Induce apoptosis	Single	-	NCT01878890	[63]
Prion disease	Cellular prion protein (PrP^C^)	Single	In vivo	-	[64]
Zika virus	nonstructural protein genes	In combination with rilpivirine and etravirine	In silico	-	[65]
Dravet syndrome	5-HT on- or off-target	Single	In vivo	-	[66]

At the moment, when efavirenz is being reconsidered as a first-line treatment for HIV-1 infection, new opportunities emerge for its use. We can consider PrEP and repurpose as the route to follow and find new services for this drug without neglecting the need to learn more about how antiretroviral drugs affect anticancer processes. Follow-up studies are required to assess drug effectiveness and its pertinence to each disease.

## 4. Conclusions

Efavirenz has followed the natural life cycle of a drug. It was first approved in the United States in 1998 and in the European Union in 1999, being a component of first-line HIV-1 treatment. Since then, a generic version has been approved, as well as a dose adjustment (Efavirenz 600 mg to 400 mg). Currently, new drugs (dolutegravir) have emerged as a better alternative to efavirenz. Nevertheless, this drug remains in the history of HIV-1 treatment and can still be applicable for new uses, such as PrEP, or for repurposing new diseases.

## Data Availability

Not applicable.
